# Methionine and S-Adenosylmethionine Regulate *Monascus* Pigments Biosynthesis in *Monascus purpureus*

**DOI:** 10.3389/fmicb.2022.921540

**Published:** 2022-06-14

**Authors:** Sheng Yin, Dongmei Yang, Yiying Zhu, Baozhu Huang

**Affiliations:** ^1^Beijing Advanced Innovation Center for Food Nutrition and Human Health, Beijing Technology and Business University, Beijing, China; ^2^Beijing Engineering and Technology Research Center of Food Additives, Beijing Technology and Business University, Beijing, China; ^3^School of Food and Health, Beijing Technology and Business University, Beijing, China

**Keywords:** methionine, S-adenosylmethionine, SAM synthetase, *Monascus* pigments, *Monascus purpureus*

## Abstract

Amino acid metabolism could exert regulatory effects on *Monascus* pigments (MPs) biosynthesis. In this work, MPs biosynthesis regulated by methionine and S-adenosylmethionine (SAM) was investigated in *Monascus purpureus* RP2. The results indicated that the addition of methionine in fermentation significantly reduced MPs production by 60–70%, and it induced a higher expression of SAM synthetase Mon2A2272 and consequently led to SAM accumulation. However, the addition of SAM in fermentation promoted MPs production by a maximum of 35%, while over-expression of the gene *Mon2A2272* led to a decrease in MPs yield, suggesting that SAM synthetase and SAM were likely to play different regulatory roles in MPs biosynthesis. Furthermore, the gene transcription profile indicated that SAM synthetase expression led to a higher expression of the transcriptional regulatory protein of the MPs biosynthesis gene cluster, while the addition of SAM gave rise to a higher expression of MPs biosynthesis activator and the global regulator LaeA, which probably accounted for changes in MPs production and the mycelium colony morphology of *M. purpureus* RP2 triggered by methionine and SAM. This work proposed a possible regulation mechanism of MPs biosynthesis by SAM metabolism from methionine. The findings provided a new perspective for a deep understanding of MPs biosynthesis regulation in *M. purpureus.*

## Introduction

*Monascus* pigments (MPs) are Chinese traditional natural colorants with an application history of thousands of years. Nowadays, they are still widely used in the fields of food, pharmaceutical, cosmetic manufacture, and printing, dyeing, and textile industries (Lin et al., 2008; Mapari et al., 2010; Feng et al., 2012; Patakova, 2013; Dufosse et al., 2014; Mostafa and Abbady, 2014; Srianta et al., 2014; Chen et al., 2015). MPs are a large class of structurally related secondary polyketide metabolites shared with a common azaphilone skeleton produced by *Monascus* spp. and are traditionally classified as red, orange, and yellow pigments (Chen et al., 2015, Chen W. et al., 2017). It is generally recognized that MPs are biosynthesized *via* the polyketide synthase (PKS) pathway, and the conserved PKS gene cluster in *Monascus* is identified to be composed of a dozen gene elements encoding the polyketide synthase, transcription factors, and other functional enzymes (Yang et al., 2014; Chen et al., 2015, Chen W. et al., 2017).

The PKS pathway responsible for MPs biosynthesis is quite complicated and still ambiguous. Continuous studies (Yang et al., 2014; Chen et al., 2015, Chen W. et al., 2017) revealed that MPs biosynthesis started with the esterification of a β-ketoacid from the fatty acid synthase pathway to the chromophore from the PKS pathway, generating the yellow intermediates. The yellow intermediates are then reduced and oxidized into the classical yellow MPs (Monascin and Ankaflavin) and orange MPs (Rubropunctatin and Monascorubrin), respectively. Finally, the classical red MPs (Rubropunctamine and Monascorubramine) are formed by direct integration of the amido group (amines or amino acids) into the orange MPs.

Based on current understanding of the MPs biosynthesis pathway, the amino donors are believed to be directly involved in the red MPs molecule formation (Chen et al., 2015, Chen W. et al., 2017). However, *in vitro* chemical reactions demonstrated that only 4 of 18 amino donors (arginine, lysine, γ-aminobutyric acid, and ammonia) directly reacted with the orange MPs to form the red MPs (Chen W. et al., 2017), indicating that not all the amino compounds could act as the precursor substrates of MPs. Even though, it is indispensable to take into account the roles of nitrogen sources in MPs biosynthesis. Several previous studies reported that different nitrogen sources supplemented in fermentation medium made a significant impact on the yield and component composition of MPs (Chen and Johns, 1993; Jung et al., 2003, 2005, 2011; Dhale et al., 2011; Hajjaj et al., 2012; Arai et al., 2013; Zhang et al., 2013; Said et al., 2014; Shi et al., 2015; Xiong et al., 2015). Therefore, it is reasonable to conclude that various nitrogen sources could exert regulatory effects on MPs biosynthesis in different ways.

Methionine is an important sulfur-containing amino acid that plays essential roles in multiple biological processes, such as DNA methylation, protein structure, and polyamine synthesis (Brosnan et al., 2007; Cavuoto and Fenech, 2012). Methionine could be transformed into S-adenosylmethionine (SAM) by the catalysis of SAM synthetase (EC 2.5.1.6) (Lieber and Lester, 2002). SAM is the major cellular methyl group donor in living organisms and it is involved in the methylation processes of DNA, RNA, proteins, metabolites, and phospholipids (Lieber and Lester, 2002; Brosnan et al., 2007; Gerke et al., 2012). In microorganisms, SAM is involved in a variety of processes, such as protection of DNA and mRNA integrity, the function of restriction enzymes, regulation of gene expression, efficient translation, cellular differentiation, stress response, and secondary metabolites biosynthesis (Lieber and Lester, 2002; Brosnan et al., 2007; Gerke et al., 2012). It is evident that the metabolism of SAM from methionine serves not only as the nitrogen source supply but also as the regulator of multiple biological processes. In this study, the bidirectional regulatory effects of methionine and SAM on MPs biosynthesis were observed in *Monascus purpureus* RP2. SAM and SAM synthetase were proved to be the key effector molecules for MPs biosynthesis regulation, which probably interacted with the global regulator LaeA and the PKS gene cluster. These findings provided new insights into the regulation mechanism of MPs biosynthesis in *M. purpureus.*

## Materials and Methods

### Strains, Plasmids, and Culture Conditions

The strains and plasmids used in this work are listed in Table 1. *Escherichia coli* strains were grown at 37°C in Luria-Bertani (LB) medium with vigorous shaking at 220 rpm in a shaking incubator with a rotational radius of 10 cm. *M. purpureus* strains were cultured in Potato Dextrose Agar (PDA) medium at 30°C. When needed, 200 μg/ml ampicillin for *E. coli* or 20 μg/ml hygromycin B for *M. purpureus* was added to the medium for recombinant screening.

**TABLE 1:** Strains and plasmids used in this work.

Strains or plasmids	Relevant features	Reference or source
**Plasmids**		
pBARGPE1-Hygro	Shuttle vector for gene expression in *E. coli* and *M. purpureus*, Amp^R^, Hygro^R^	Laboratory collection
pBARGPE1-2272	SAM synthetase gene *Mon2A2272* cloned in pBARGPE1-Hygro, Amp^R^, Hygro^R^	This work
**Strains**		
*E. coli* DH5α	Host for cloning	TIANGEN, Beijing, China
*E. coli* 2272	*E. coli* DH5α harboring pBARGPE1-2272, Amp^R^	This work
*M. purpureus* RP2	Wild strain; Donor of SAM synthetase gene *Mon2A2272*	Laboratory collection
*M. purpureus* 2272	*M. purpureus* RP2 harboring pBARGPE1-2272, Hygro^R^	This work

For MPs production by liquid-state fermentation, *M. purpureus* was cultured at 30°C in PDA medium for 10 days and the spore suspension was prepared by washing the mycelium with sterile water and collected by filtration with sterile gauze. The spore suspension (10%) was inoculated into seed medium (40 g/L rice flour, 8 g/L peptones, 5 g/L soybean meal, 1 g/L MgSO_4_⋅7H_2_O, 2 g/L KH_2_PO_4_, and 2 g/L NaNO_3_) and cultured at 33°C with vigorous shaking at 200 rpm in a shaking incubator with a rotational radius of 10 cm for 48 h. The seed culture (10%) was then inoculated into a fermentation medium (20 g/L glucose, 5 g/L Yeast Nitrogen Base W/O Amino acids, 5 g/L K_2_HPO_4_⋅3H_2_O, 0.5 g/L MgSO_4_⋅7H_2_O, 5 g/L KH_2_PO_4_, 0.1 g/L CaCl_2_, 0.03 g/L MnSO_4_⋅H_2_O, 0.01 g/L FeSO_4_⋅7H_2_O, and 0.01 g/L ZnSO_4_⋅7H_2_O) and cultured at 33°C with vigorous shaking at 200 rpm in a shaking incubator with a rotational radius of 10 cm for 12 days. When needed, 3 g/L methionine or 1 g/L SAM was added to the fermentation medium. For colony morphology observation, *M. purpureus* was cultured at 30°C in the fermentation medium containing agar (20 g/L) for 6–9 days.

### Measurement of *Monascus* Pigments Production

For measurement of MPs production, the fermentation culture was centrifuged at 8,000 × *g* for 10 min to collect mycelia and medium supernatant, respectively. Samples of the mycelia or medium supernatant were soaked in 50 ml of 70% (v/v) ethanol and incubated in a water bath (60°C) for 1 h to extract MPs. The extraction mixture was centrifuged at 8,000 × *g* for 10 min to collect the supernatant, which was used to measure MPs concentration by spectrophotometer (OD 410 nm for yellow MPs, OD 468 nm for orange MPs, and OD 505 nm for red MPs).

### DNA Manipulation Techniques

Standard DNA manipulation techniques were performed as described by Green and Sambrook (2012). Total RNA from *M. purpureus* was prepared using the RNAprep Pure Plant Kit (TIANGEN, Beijing, China) following the manufacturer’s instructions. RNA was subjected to reverse transcription to generate cDNA using the Quantscript RT Kit (TIANGEN, Beijing, China) following the manufacturer’s protocol. Quantitative Real-Time PCR (q RT-PCR) was performed using the SuperReal PreMix Plus (SYBR Green) Kit (TIANGEN, Beijing, China) in the CFX96 Touch Real-Time PCR System (Bio-Rad, United States) with the following cycling conditions: 95°C for 2 min, followed by 40 cycles of 94°C for 20 s, 63°C for 45 s, and 60°C for 5 min. The *GAPDH* gene was used for transcript normalization. All reactions were performed in triplicate. The 2^–ΔΔCt^ method was used to analyze the data, which was corrected for primer efficiencies using the untreated group means as the reference condition (Schmittgen and Livak, 2008; Putt et al., 2017).

DNA amplification and recombinant DNA construction were performed using the Ex Taq DNA Polymerase and DNA Ligation Kit (Takara, Beijing, China) following the manufacturer’s protocol. Plasmid DNA from *E. coli* was isolated using the High-purity Plasmid Miniprep Kit (TIANGEN, Beijing, China) according to the manufacturer’s instructions. A standard heat shock transformation method was used to introduce plasmid DNA to *E. coli* (Green and Sambrook, 2012). Primers used for gene transcription level assay by q RT-PCR and gene cloning by PCR are listed in Supplementary Table 1.

### Vector Construction for Over-Expression of the S-Adenosylmethionine Synthetase Gene *Mon2A2272*

The SAM synthetase gene *Mon2A2272* was amplified by PCR from the cDNA derived from the total RNA of *M. purpureus* RP2 using the specific primers SAMS-F/R designed according to the genomic sequence of *M. purpureus* RP2. The amplicon was inserted into the shuttle vector pBARGPE1-Hygro for gene expression in *E. coli* and *M. purpureus* to construct the recombinant vector pBARGPE1-2272. The DNA ligation mixture was transformed into *E. coli* DH5α and transformants were screened on LB agar plates containing ampicillin. The recombinant vector was verified by sequencing and alignment analysis using the DNAMAN software package and BLAST Program at NCBI against the GenBank database.

### Electroporation of *Monascus purpureus*

The recombinant vector pBARGPE1-2272 was introduced into *M. purpureus* RP2 by electroporation as previously described with modifications (Kim et al., 2003; Lakrod et al., 2003). For the preparation of the electrocompetent protoplast of *M. purpureus* RP2, a fresh spore suspension was prepared as described previously and was spread on a PDA plate and cultured at 30°C for 30–40 h. Mycelia (300 mg) were collected and washed with 1 M MgSO_4_ and incubated at 30°C for 3 h in 50 ml of enzymolysis buffer (1% snailase, 0.1% cellulase, and 0.3% lysozyme). The enzymolysis solution was filtered with sterile gauze and the spheroplast suspension was collected and centrifuged at 8,000 × *g* at 4°C for 5 min. The spheroplasts were harvested and washed with cooled sorbitol buffer (1 M) and resuspended in 200 μl of the cooled protoplast buffer (25 mM Tris-HCl, pH 7.5; 25 mM CaCl_2_, 1.2 M sorbitol).

For electroporation of *M. purpureus* RP2, the purified recombinant plasmid pBARGPE1-2272 (1 μg) and the cooled protoplast suspension (100 μl, 10^9^ protoplasts/ml) were mixed well and transferred to a cooled electroporation cuvette (Bio-Rad, United States) with a 0.2-cm electrode gap. The electroporation cuvette was incubated in an ice bath for 15 min and electroporated in Gene Pulser (Bio-Rad, United States) using the following settings: voltage, 3 kV/cm; capacitance, 25 μF; and resistance, 400 Ω. Following the electric pulse, 1 ml of regeneration broth (Potato Dextrose Broth plus 1.2 M sorbitol) was immediately added to the cuvette and the electroporation mixture was transferred to a sterile 1.5 ml tube and incubated with agitation at 1,000 × *g* at 30°C for 2 h. The mixture was plated onto PDA plates containing hygromycin B and cultured at 30°C in the dark for 3–6 days. The recombinant strain *M. purpureus* 2272 was screened and validated by colony PCR of the hygromycin resistance gene using the specific primers Hyg-F/R. Additionally, q RT-PCR was carried out for analysis of the transcriptional level of *Mon2A2272* in *M. purpureus* 2272.

### Measurement of S-Adenosylmethionine

For measurement of SAM produced by *M. purpureus*, the fermentation culture was centrifuged at 8,000 × *g* for 10 min to collect mycelia and medium supernatant, respectively. The medium supernatant was filtered with a 0.22 μm pore membrane and used for SAM determination. The wet mycelia were dried at −80°C by vacuum freezing and incubated in the solution containing 4 ml of 1.5 M perchloric acid and 4 ml of 1.5 M ammonia water at 4°C for overnight. The mixture was centrifuged at 10,000 × *g* for 15 min to collect the supernatant for SAM determination. SAM was determined by high-performance liquid chromatography (HPLC) (SHIMADZU, Shanghai, China) as previously described with modifications (Wang et al., 2012). The HPLC analysis conditions were as follows: Inertsil ODS-3 column (4.6 mm × 250 mm, 5 μm, SHIMADZU, Shanghai, China); injection volume 20 μl; oven temperature 30°C; flow rate 1 ml/min; the eluate was monitored at 260 nm. Mobile phase A (10 mM ammonium formate buffer, pH 3.5) and phase B (methanol) were run on an isocratic elution program (95% phase A buffer and 5% phase B buffer). The standard chemicals SAM was purchased from Macklin Biochemical Co., Ltd (Shanghai, China).

## Results and Discussion

### Addition of Methionine Reduced *Monascus* Pigments Production and Enhanced S-Adenosylmethionine Synthetase Expression and S-Adenosylmethionine Generation

**FIGURE 1:**
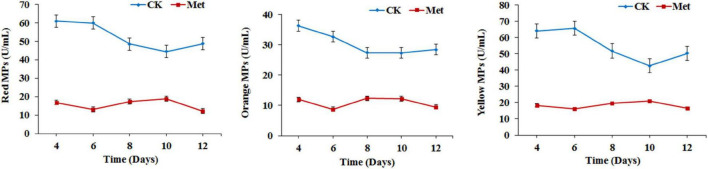
*Monascus* pigments (MPs) production in *Monascus purpureus* RP2. CK, fermentation without addition of methionine; Met, fermentation with addition of 3 g/L methionine.

**FIGURE 2:**
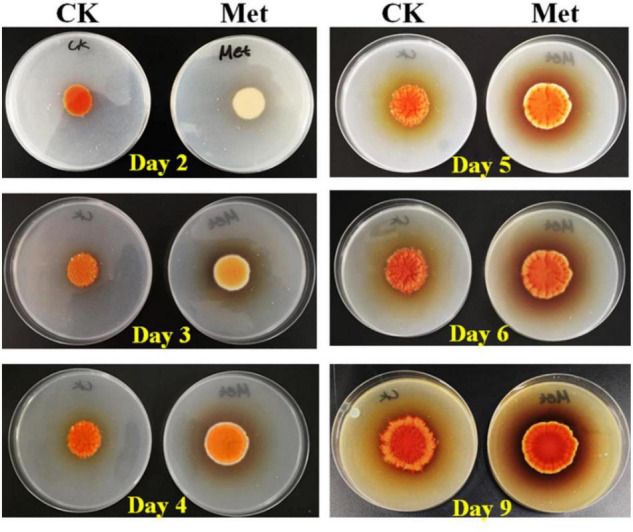
Cultivation of *M. purpureus* RP2 in fermentation agar plates. CK, fermentation without addition of methionine; Met, fermentation with addition of 3 g/L methionine.

The fermentation medium containing YNB without amino acids as the nitrogen source was used to evaluate the effect of methionine addition on MPs biosynthesis in *M. purpureus* RP2. Evaluation of serial gradient concentrations (1–9 g/L) of methionine indicated that 3 g/L was the optimal volume of addition that exerted a significant effect on MPs production (data not shown). Fermentation results showed that the yields of red, orange, and yellow MPs were all significantly reduced by 60–70% during 12 days of liquid fermentation with the addition of 3 g/L methionine (Figure 1). Meanwhile, the bright red, orange, and yellow colors of MPs became much more intense within and around the mycelium colony cultivated without methionine than with methionine in the fermentation agar plates (Figure 2). MPs production was remarkably inhibited in the presence of methionine in both liquid fermentation and plate culture. In addition, it is noteworthy that the morphologies were quite different between the mycelium colonies grown in plates with and without methionine as shown in Figure 2, indicating that the addition of methionine also changed the mycelial development of *M. purpureus* RP2.

To figure out the reason that methionine inhibited MPs production, the metabolism of methionine was investigated in *M. purpureus* RP2. Generally, methionine could be transformed to SAM by the catalysis of SAM synthetase and the derivative would act as the major cellular methyl donor and precursor to organic radicals involved in a variety of important biological processes (Lieber and Lester, 2002; Brosnan et al., 2007). Unsurprisingly, it was detected that the concentration of intracellular SAM in *M. purpureus* RP2 increased by 100–200 μg/ml in fermentation with methionine compared with that without methionine. In addition, q RT-PCR analysis also showed that the transcriptional level of the SAM synthetase gene *Mon2A2272* in *M. purpureus* RP2 at the beginning of fermentation was about 12-fold higher with methionine than without methionine, which could account for SAM generation. Therefore, it is reasonable to conclude that the addition of methionine in the fermentation medium induced SAM synthetase Mon2A2272 expression and consequently enhanced SAM generation, directly or indirectly leading to a significant reduction in MPs production in *M. purpureus* RP2. However, it is still questionable whether SAM synthetase Mon2A2272 expressed at a higher level or SAM accumulation acted as the effector that affected MP production.

### Addition of S-Adenosylmethionine Enhanced *Monascus* Pigments Production

**FIGURE 3:**
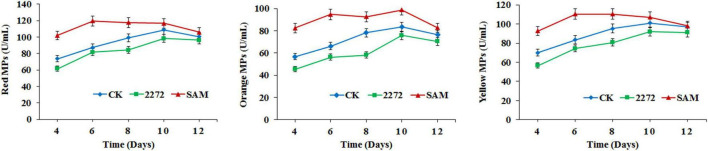
*Monascus* pigments production in *M. purpureus* RP2 and *M. purpureus* 2272. CK, *M. purpureus* RP2 fermentation without addition of amino acid; 2272, *M. purpureus* 2272 fermentation without addition of amino acid; (SAM), *M. purpureus* RP2 fermentation with addition of 1 g/L SAM.

**FIGURE 4:**
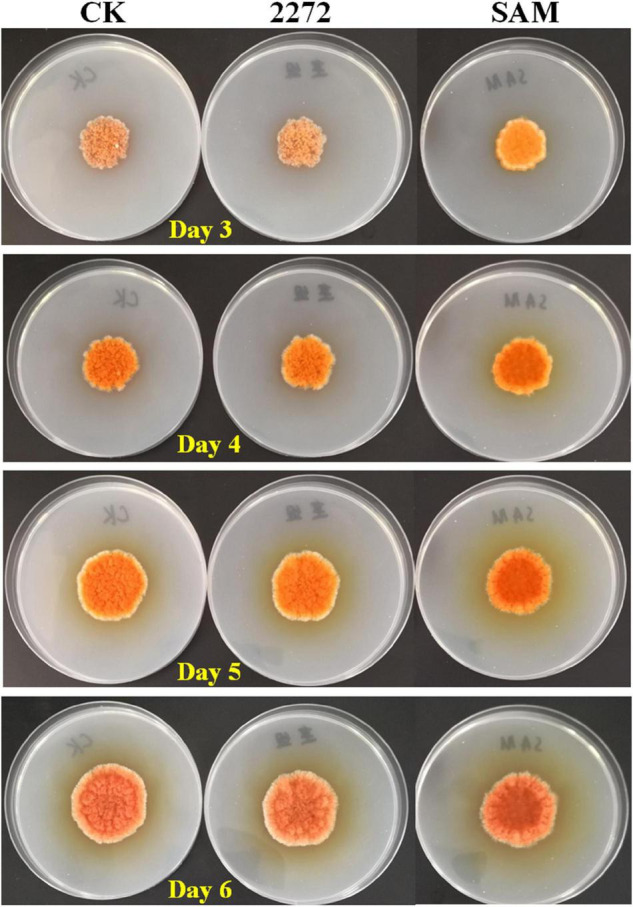
Cultivation of *M. purpureus* RP2 and *M. purpureus* 2272 in fermentation agar plates. CK, *M. purpureus* RP2 fermentation without addition of amino acid; 2272, *M. purpureus* 2272 fermentation without addition of amino acid; SAM, *M. purpureus* RP2 fermentation with addition of 1 g/L SAM.

**FIGURE 5:**
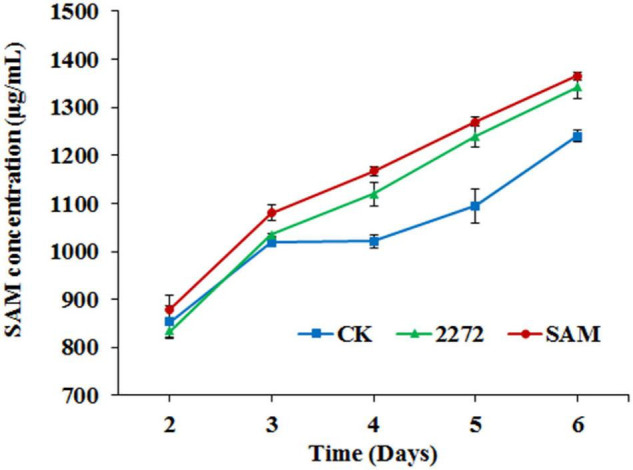
S-adenosylmethionine accumulation in the fermentation broth of *M. purpureus* RP2 and *M. purpureus* 2272. CK, *M. purpureus* RP2 fermentation without addition of amino acid; 2272, *M. purpureus* 2272 fermentation without addition of amino acid; SAM, *M. purpureus* RP2 fermentation with the addition of 1 g/L SAM.

**FIGURE 6:**
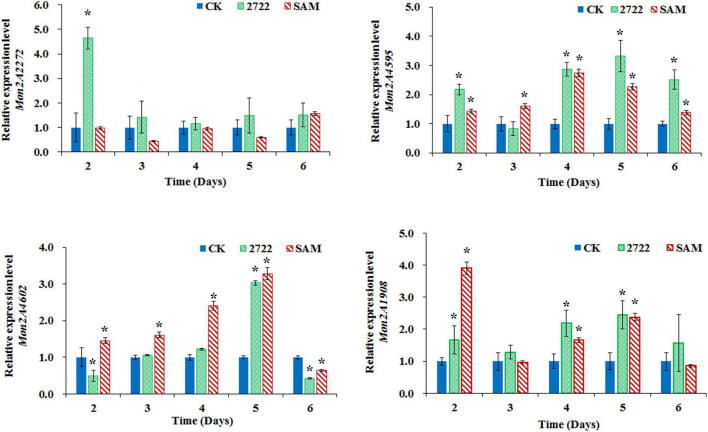
Gene transcriptional level assay in *M. purpureus* RP2 and *M. purpureus* 2272. CK, *M. purpureus* RP2 fermentation without addition of amino acid; 2272, *M. purpureus* 2272 fermentation without addition of amino acid; SAM, *M. purpureus* RP2 fermentation with the addition of 1 g/L SAM; Bars with an asterisk are significantly different (*p* < 0.05).

As the addition of methionine led to SAM generation and MP production reduction in *M. purpureus* RP2, it is essential to reveal the causal connection between SAM accumulation and MP production. The fermentation with the addition of 1 g/L SAM was conducted to investigate the effect of high-level SAM on MP production in *M. purpureus* RP2. Unexpectedly, a maximum 35% increase in MPs production was detected in fermentation with SAM in comparison with that without SAM (Figure 3). The fermentation agar plate cultivation also showed that a brighter yellow or orange halo was observed around the mycelium colony with SAM (Figure 4), which was in accordance with MPs yield determination. Moreover, the addition of SAM also altered the morphology of the mycelium colony (Figure 4), but it is a different pattern from that derived from the addition of methionine (Figure 2). In addition, though the addition of SAM resulted in a higher accumulation of SAM in *M. purpureus* RP2 after 3 days of fermentation as shown in Figure 5, q RT-PCR assay indicated that extra SAM did not affect the expression of the SAM synthetase gene *Mon2A2272* (Figure 6). It suggested that SAM individually played the regulatory role in MP production and mycelium development in *M. purpureus* RP2.

### S-Adenosylmethionine Synthetase Mon2A2272 Over-Expression Enhanced S-Adenosylmethionine Generation and Reduced *Monascus* Pigments Production

As previously reported, the addition of methionine induced SAM synthetase Mon2A2272 expression, enhanced SAM generation, and inhibited MPs production. SAM synthetase or SAM was suspected to be the effector of the reduction in MP production. However, when a high concentration of SAM was added in fermentation, MP production increased as shown in Figures 3, 4. Hence, the causal connection between the high expression level of SAM synthetase and MPs production was further investigated by the construction of the recombinant strain *M. purpureus* 2272 with over-expression of the SAM synthetase gene *Mon2A2272*. The SAM synthetase gene *Mon2A2272* cloned from *M. purpureus* RP2 was 1,161 bp in size and encoded a protein containing 386 amino acid residues, which shared a 100% homology with the SAM synthase from *M. purpureus* (GenBank Accession No. TQB68059.1). The q RT-PCR analysis showed that the transcriptional level of the SAM synthetase gene *Mon2A2272* in *M. purpureus* 2272 was about 4.5-fold higher than that in the wild strain *M. purpureus* RP2 on day 2 of fermentation (Figure 6). Consequently, much more SAM was generated and accumulated in the fermentation broth in *M. purpureus* 2272 than that in *M. purpureus* RP2 after 3 days of fermentation (Figure 5). In addition, the yields of red, orange, and yellow MPs all decreased in *M. purpureus* 2272 in comparison with *M. purpureus* RP2 during 12 days of fermentation (Figure 3). Morphology observation showed no obvious difference between the mycelium colonies of *M. purpureus* 2272 and *M. purpureus* RP2 (Figure 4), indicating that expression of the SAM synthetase gene *Mon2A2272* did not affect the mycelial development. The results gave a probable explanation for MPs reduction by the addition of methionine in fermentation. SAM synthetase expression likely exerted the negative regulation on MPs production in *M. purpureus* RP2.

### S-Adenosylmethionine and S-Adenosylmethionine Synthetase Mon2A2272 Both Regulated *Monascus* Pigments Biosynthesis Gene Cluster Expression

The conserved PKS gene cluster was responsible for MPs biosynthesis in *Monascus* (Yang et al., 2014; Chen et al., 2015, Chen W. et al., 2017). A classical PKS gene cluster was detected in *M. purpureus* RP2 and it consisted of 15 structural genes encoding functional enzymes and two regulatory elements *Mon2A4595* and *Mon2A4602* (Supplementary Figure 2). *Mon2A4595* encoded the transcriptional regulatory protein and shared a 99% homology with the transcription factor MpigI (GenBank Accession No. APZ73944.1) from *Monascus ruber* M7; and *Mon2A4602* encoded the pigment biosynthesis activator and shared a 97% homology with the pigment biosynthesis activator PigR (GenBank Accession No. AGL44390.1) from *M. ruber* M7. Yang et al. (2014) reported that most genes in the PKS cluster including the two regulatory elements were expressed at higher levels under high pigment production conditions in *M. purpureus* YY-1 and Chen D. et al. (2017) reported that expression of the transcriptional regulatory protein in the PKS cluster was upregulated when pigment biosynthesis was induced by blue light in *M. purpureus* M9. Hence, the MPs biosynthesis regulatory elements *Mon2A4595* and *Mon2A4602* were selected for gene transcriptional level analysis by qRT-PCR during fermentation. As shown in Figure 6, the transcriptional levels of *Mon2A4595* and *Mon2A4602* both increased in *M. purpureus* RP2 with addition of SAM and in *M. purpureus* 2272 with an expression of SAM synthetase Mon2A2272 during the early 6 days of fermentation when MPs was synthesized in large amounts. Generally, SAM synthetase Mon2A2272 expression led to a higher expression of the transcriptional regulatory protein Mon2A4595, while the addition of SAM gave rise to a higher expression of the pigment biosynthesis activator Mon2A4602. Furthermore, the addition of SAM in fermentation resulted in higher expressions of most functional enzymes encoding genes in the PKS cluster in comparison with that without SAM addition and with Mon2A2272 expression (Supplementary Figure 3). This could probably explain the difference in MP production yield between *M. purpureus* RP2 with and without the addition of SAM and *M. purpureus* 2272 with the expression of SAM synthetase Mon2A2272. It suggested that SAM and SAM synthetase both could affect the expression of the PKS gene cluster by the regulatory elements to regulate MPs biosynthesis.

Furthermore, the global regulator, Mon2A1908 that shared a 100% homology with the global regulator of sporulation and secondary metabolism LaeA (GenBank Accession No. AIY63188.1) from *M. ruber* M7 was subjected to transcription analysis in response to the addition of SAM and expression of SAM synthetase Mon2A2272. It is observed that the transcriptional level of *Mon2A1908* was about 4-fold higher in *M. purpureus* RP2 with the addition of SAM than that in *M. purpureus* RP2 without SAM and *M. purpureus* 2272 at the beginning of fermentation (Figure 6). The biosynthesis of many secondary metabolites in filamentous fungi is regulated by global transcriptional regulators, such as LaeA (Lind et al., 2018). In *Aspergillus* species, the global regulatory proteins VeA and LaeA have been shown to control both development and secondary metabolism (Calvo and Cary, 2015). Liu et al. (2016) reported that inactivation of LaeA in *M. ruber* drastically reduced the production of multiple secondary metabolites, such as *Monascus* pigments and citrinin and also resulted in the formation of an abnormal colony phenotype with abundant aerial hyphae. As previously reported, the addition of SAM enhanced MPs production and altered the morphology of the mycelium colony of *M. purpureus* RP2, it is reasonable to conclude that SAM probably regulates MPs biosynthesis and the mycelial development by interaction with the global regulator Mon2A1908.

Secondary metabolites biosynthesis coupled with asexual and sexual development in *Aspergillus* fungi is regulated by various environmental signals, such as temperature, pH, light, and carbon or nitrogen sources (Calvo et al., 2002; Calvo and Cary, 2015; Liu et al., 2016; Lind et al., 2018). In addition, it is controlled by a complex global network involved multiple regulators at the cellular level (Calvo et al., 2002; Calvo and Cary, 2015; Lind et al., 2018). This work proposed a possible bi-directional control mechanism of MPs biosynthesis by SAM metabolism from methionine in *M. purpureus* RP2. It is interesting to note that methionine could induce expression of SAM synthetase and SAM generation and exhibit the inhibited effect on MPs production, while high-level SAM inversely enhanced MPs biosynthesis. It is possible that the dynamic change in SAM synthetase expression level and the pool of SAM would be important signals of the regulatory network for MPs biosynthesis. Nevertheless, it is indispensable to dig out more details on the molecule components and their interactions involved in the regulatory network triggered by methionine and SAM. The findings in this work would provide a new perspective for a deep understanding of MPs biosynthesis regulation in *M. purpureus.*

## Data Availability Statement

The datasets presented in this study can be found in online repositories. The names of the repository/repositories and accession number(s) can be found in the article/Supplementary Material.

## Author Contributions

SY conceived and designed the experiments, wrote the manuscript, contributed to the manuscript revision, financial support, and supervision. DY, YZ, and BH performed the experimental work and data analysis. All authors contributed to the article and approved the submitted version.

## Conflict of Interest

The authors declare that the research was conducted in the absence of any commercial or financial relationships that could be construed as a potential conflict of interest.

## Publisher’s Note

All claims expressed in this article are solely those of the authors and do not necessarily represent those of their affiliated organizations, or those of the publisher, the editors and the reviewers. Any product that may be evaluated in this article, or claim that may be made by its manufacturer, is not guaranteed or endorsed by the publisher.
