# Supplementary material for: Methionine and S-Adenosylmethionine Regulate Monascus Pigments Biosynthesis in Monascus purpureus
Source: Front Microbiol. 2022 Jun 14;13:921540. doi: 10.3389/fmicb.2022.921540 (PMC9237499; doi:10.3389/fmicb.2022.921540)
Supplement: Supplementary file 1 [file Data_Sheet_1.DOCX]

Supplementary Material

# Supplementary Figures


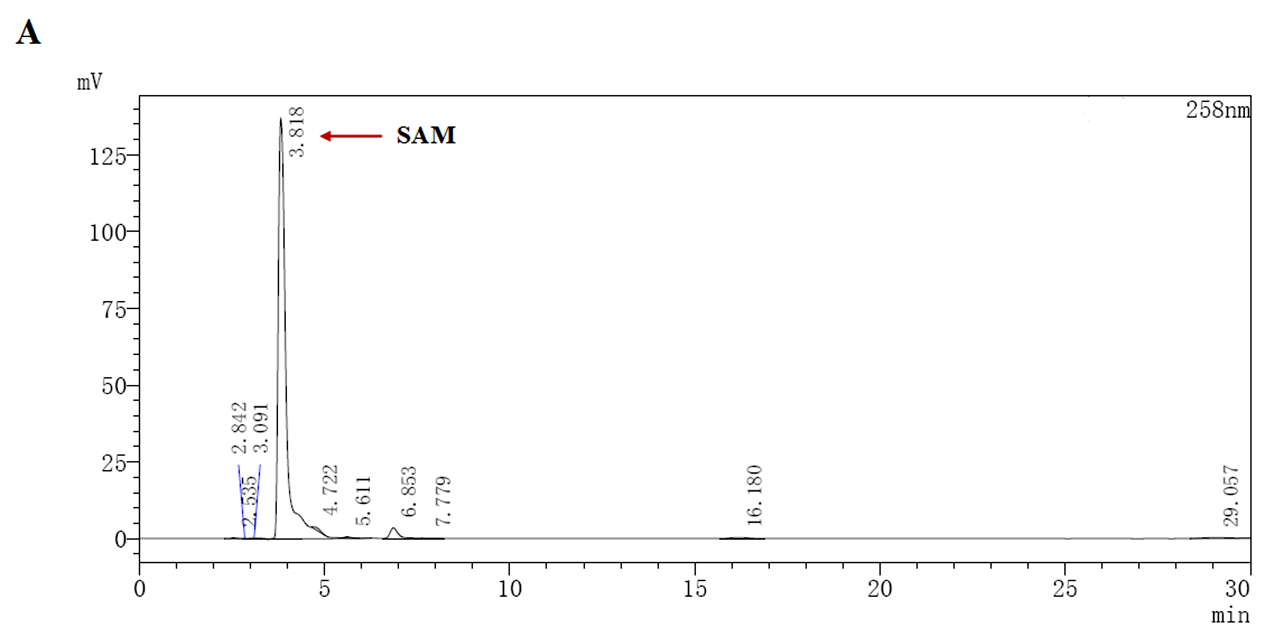

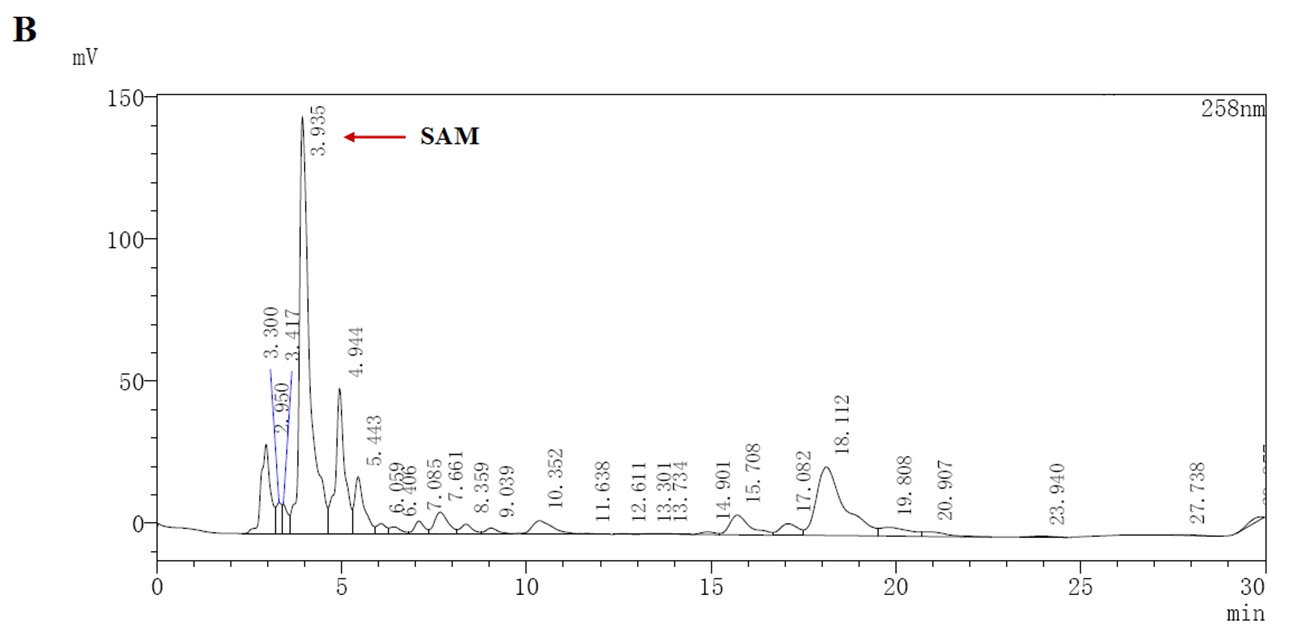


**Supplementary Figure 1. SAM detection of the standard sample (A) and the sample of *M. purpureus* fermentation (B) by HPLC analysis**


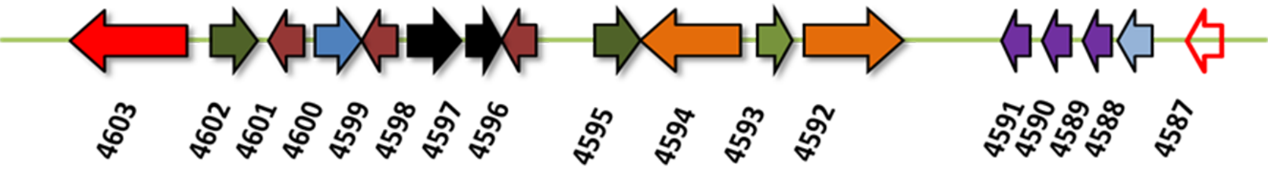


**Supplementary Figure 2. MPs biosynthesis PKS cluster of *M. purpureus* RP2**

4587 (Mon2A4587), MFS multidrug transporter (98% identity with GenBank No. ANS12245.1);

4588 (Mon2A4588), decarboxylase (100% identity with GenBank No. ANS12244.1);

4589 (Mon2A4589), deacetylase (100% identity with GenBank No. QGA67179.1);

4590 (Mon2A4590), monooxygenase (100% identity with GenBank No. ALT31754.1);

4591 (Mon2A4591), acyltransferase (100% identity with GenBank No. QGA67183.1);

4592 (Mon2A4592), fatty acid synthase β subunit (99% identity with GenBank No. AGL44430.1);

4593 (Mon2A4593), hypothetical protein (100% identity with GenBank No. TQB69636.1);

4594 (Mon2A4594), fatty acid synthase α subunit (100% identity with GenBank No. QGA67185.1);

4595 (Mon2A4595), transcriptional regulatory protein (99% identity with GenBank No. APZ73944.1);

4596 (Mon2A4596), reductase (100% identity with GenBank No. APZ73943.1);

4597 (Mon2A4597), serine hydrolase (100% identity with GenBank No. APZ73942.1);

4598 (Mon2A4598), FAD dependent dehydrogenase (100% identity with GenBank No. APZ73941.1);

4599 (Mon2A4599), oxidoreductase (100% identity with GenBank No. AHA93896.1);

4600 (Mon2A4600), 3-O-acetyltransferase (100% identity with GenBank No. AGI63864.1);

4601 (Mon2A4601), short-chain alcohol dehydrogenase (100% identity with GenBank No. AGI63865.1);

4602 (Mon2A4602), pigment biosynthesis activator (99% identity with GenBank No. QGA67219.1);

4603 (Mon2A4603), polyketide synthase (99% identity with GenBank No. ALN44200.1).


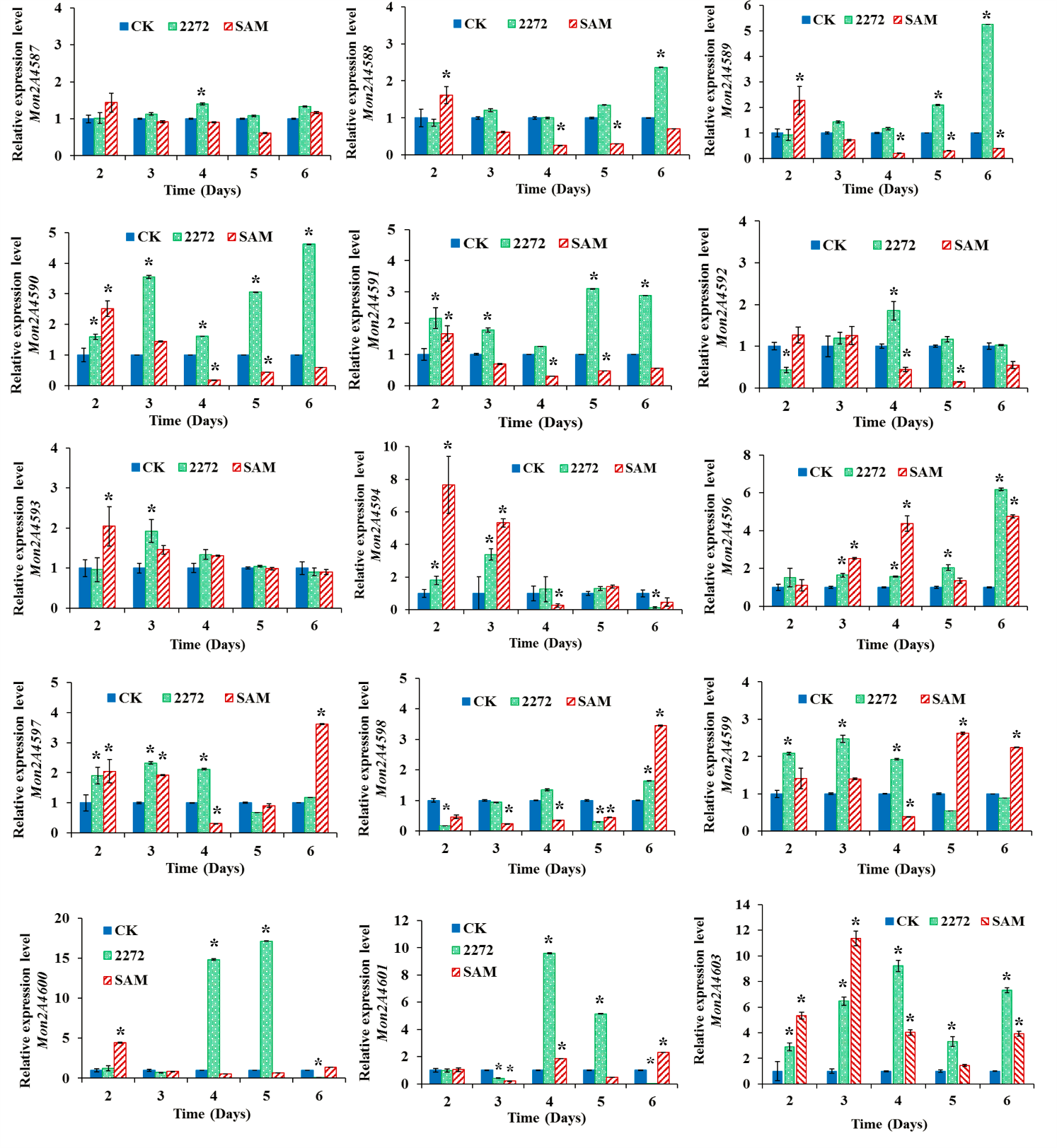


**Supplementary Figure 3. Gene transcriptional level assay of MPs biosynthesis PKS cluster in *M. purpureus* RP2 and *M. purpureus* 2272**

CK, *M. purpureus* RP2 fermentation without addition of amino acid; 2272, *M. purpureus* 2272 fermentation without addition of amino acid; SAM, *M. purpureus* RP2 fermentation with addition of 1 g/L SAM; Bars with asterisk are significantly different (P < 0.05).

# Supplementary Table

**Supplementary Table 1 Primers used in this work**

| Gene | Primer | Sequence (5’-3’) |
| --- | --- | --- |
|  | Quantitative Real-Time PCR |  |
| *Mon2A4603* | 4603-F | AGATTACTGTCGTTGAAG |
|  | 4603-R | CTCTCAGGATACTCTCAT |
| *Mon2A4602* | 4602-F | GTTCTGGATGGCGTATAG |
|  | 4602-R | ACCTGTTCGTTCAATGTC |
| *Mon2A4601* | 4601-F | ATACCATTGTCTATCTCAC |
|  | 4601-R | GATTTCGTCTTTCATAGC |
| *Mon2A4600* | 4600-F | TTGATGTCTTGATCTGTC |
|  | 4600-R | CTATATACTCCGCATACG |
| *Mon2A4599* | 4599-F | TACTGGAACGATGCCTAC |
|  | 4599-R | CACTTCGGTCAGGTTATAC |
| *Mon2A4598* | 4598-F | AATACAGCCTCCAGAAGA |
|  | 4598-R | TGTTCATCACCGTAATGG |
| *Mon2A4597* | 4597-F | AAGACGAAGGATGTTGTG |
|  | 4597-R | TCAAATCCGTCTCTGGAG |
| *Mon2A4596* | 4596-F | ATCATCTCGTTCAAGTTCAAG |
|  | 4596-R | GATGCCGTTGTGATTGAC |
| *Mon2A4595* | 4595-F | TGTGTACTCTCTCCCTCTC |
|  | 4595-R | ATTCCGACGACATTGCTAT |
| *Mon2A4594* | 4594-F | CTCCTGATTGAACTACTT |
|  | 4594-R | ATATCTCTGGATGGTCTC |
| *Mon2A4593* | 4593-F | CGATACTGGACGGGTTCT |
|  | 4593-R | CGTTGCCATTGTAGTTGAC |
| *Mon2A4592* | 4592-F | ATTATCAAGCGACTCAAT |
|  | 4592-R | TCAATGTATGTCATCTCAG |
| *Mon2A4591* | 4591-F | AACTTCAATGAGGCATATTATG |
|  | 4591-R | TAGTTCCACGCTCAATTC |
| *Mon2A4590* | 4590-F | AAGTCTATTGGCGTTCTC |
|  | 4590-R | GTCTACGTCTATAGTCTTCAG |
| *Mon2A4589* | 4589-F | GAGAAGAACGTCCGCTTG |
|  | 4589-R | AACTCCAGCAGCAACTTC |
| *Mon2A4588* | 4588-F | TACATTGCCAGTTTACAG |
|  | 4588-R | GTATGGAGGATTAGTTCAC |
| *Mon2A4587* | 4587-F | TGGCATCTTACTATCTCC |
|  | 4587-R | GAATGTAGACTCCACTCA |
| *Mon2A2272* | 2272-F | GAGACTGCTACCAAGACT |
|  | 2272-R | CATCACGGATAACCTTCTG |
| *Mon2A1908* | 1908-F | ACTCGTAGCGGATGTAAGA |
|  | 1908-R | CCGTGCTTGGTAGATGTG |
| *GAPDH* | GAPDH-F | CCGTATTGTCTTCCGTAAC |
|  | GAPDH-R | GTGGGTGCTGTCATACTTG |
|  | PCR |  |
| *Mon2A2272* | SAMS-F | GAATTCATGGGCAGCACTGCTTCG |
|  | SAMS-R | GGTACCCTAGAATTTGAGGGCCTTGG |
| Hygromycin resistance gene | Hyg-F | ATCGTTATGTTTATCGGCACT |
|  | Hyg-R | TGGCGACCTCGTATTGG |
